# Synergistic Beneficial Effects of Flaxseed ( *Linum usitatissimum* L.) Oil and Olive ( *Olea europaea* L.) Oil Against Metabolic Dysfunction Associated Fatty Liver and Its Complications

**DOI:** 10.1002/fsn3.4638

**Published:** 2024-12-02

**Authors:** Sana Noreen, Bushra Hashmi, Tabussam Tufail, Ali Ikram, Muhammad Tayyab Arshad, Kodjo Théodore Gnedeka

**Affiliations:** ^1^ University Institute of Diet and Nutritional Sciences The University of Lahore Lahore Pakistan; ^2^ University Institute of Food Science and Technology The University of Lahore Lahore Pakistan; ^3^ Togo Laboratory: Applied Agricultural Economics Research Team (ERE2A) University of Lomé Lomé Togo

**Keywords:** ALA, flaxseed, MAFLD, olive

## Abstract

Flaxseed and olive oil effectively treat numerous diseases and health conditions, particularly metabolic disorders. Traditional medicine has used both oils for managing cardiovascular disease, diabetes, gastrointestinal dysfunctions, metabolic‐dysfunction‐associated fatty liver disease (MAFLD), obesity, and more. This review explores the bioactive and polyphenolic compounds in flaxseed and olive oils that provide anti‐inflammatory, antioxidant, anti‐microbial, hepatoprotective, cardioprotective, antidiabetic, and gastroprotective benefits. Flaxseed oil contains beneficial compounds like alpha‐linolenic acid (ALA), lignans, ferulic acid, p‐coumaric acid, and phytosterols. It contributes to its therapeutic effects on fatty liver disease and other conditions. Olive oil contains phenolic compounds, including oleic acid, hydroxytyrosol, and tocopherols, which are similarly linked to metabolic health benefits, especially in managing MAFLD. The purpose of this review is to elucidate the mechanisms of action of these bioactive compounds, highlighting their potential in managing various metabolic diseases.

## Graphical abstract

In this review, we discuss the bioactive compounds in flaxseed and olive oils, notably their polyphenols and fatty acids, and highlight their anti‐inflammatory, antioxidant, hepatoprotective, cardioprotective, antidiabetic, and gastroprotective effects. Our analysis focuses on the individual and synergistic effects of flaxseed's alpha‐linolenic acid (ALA), lignans, and phytosterols alongside olive oil's phenolic compounds such as oleic acid, hydroxytyrosol, and tocopherols. We also elucidate the biochemical mechanisms by which these compounds mitigate metabolic dysfunction, particularly in metabolic‐dysfunction‐associated fatty liver disease (NAFLD).

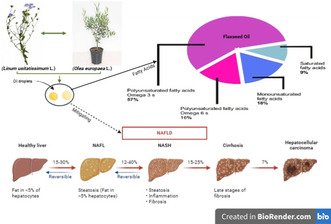

## 1 Introduction

One of the leading reasons for various diseases is a manageable inflammatory response. Different polyphenolic compounds, flavonoids, alkaloids, and other phenolic compounds have anti‐inflammatory properties in medicinal herbs and plants. Utilizing these herbs to treat illness is economically friendly and reduces public health burden (Shahrajabian 2021; Al Juhaimi et al. 2017). Resveratrol is a natural polyphenol found in grapes with beneficial properties like improving insulin resistance and maintaining ethanol metabolism (Farhan and Rizvi 2023). Anthocyanins are a group of flavonoids that reduce the progression of metabolic (dysfunction) associated fatty liver disease (MAFLD/NAFLD) and insulin resistance (Ding et al. 2021). Lycopene is a liposoluble carotenoid extracted from tomatoes and can reduce oxidative stress and inflammation and maintain metabolic processes (Rocha et al. 2023). Catechins, a polyphenolic compound extracted from tea, have various benefits, such as regulating metabolic tissue functions, improving oxidative stress, inflammation, insulin resistance, and lipid oxidation (Hodges, Sasaki, and Bruno 2020). Bioactive compounds in coffee, like caffeic acid and trigonelline, can improve liver triglycerides metabolism, fibrotic status, and oxidative stress in patients with fatty liver disease (Li et al. 2021). Recent studies show that *Moringa oleifera* leaves help to improve insulin resistance, liver metabolic diseases, and modified gene expression of MAFLD (Monraz‐Méndez et al. 2022). Avocatin B (AvoB) derived from avocado inhibits oxidation and improves insulin resistance. Daily consumption of avocados results in significant MAFLD. Functional foods protect against metabolic problems and weight management. By 2025, their growth rate in food markets is near to increase by 6.8%, increasing the production of herbal products (Sandner et al. 2020). Despite being small parts of fruits, they work well with sugars to enhance the sensory experience and final fruit quality of both raw and processed fruits (Arslan and Özcan 2011).

Metabolic dysfunction‐associated fatty liver disease (MAFLD) is a sequence of multiple diseases associated with the deposition of enormous fat in the liver. It is a complex of metabolic disorders in which numerous factors affect its progression such as genetics, dietary habits, environment, gut microbiota, immunity, metabolism, unhealthy lifestyle, and many other factors (Rong et al. 2023). It has an association with Type 2 diabetes which contributes to the risk of cirrhosis and other complications. Advanced liver fibrosis is a basis of prognostic marker for liver‐related diseases and a comprehensive cause of mortality. It can be evaluated with a junction of non‐invasive tests (Powell, Wong, and Rinella 2021). Recently, experts have examined the fact that MAFLD does not support the present knowledge, and they proposed metabolic (dysfunction) associated fatty liver disease (MAFLD) as a more appropriate term (Xian, Weng, and Xu 2021). MAFLD is diagnosed in patients with hepatic steatosis having three metabolic conditions such as obesity, Type 2 diabetes mellitus (T2DM), and metabolic dysregulation in individuals. This point of reference allows clinicians to pick out more patients at risk of unfavorable outcomes. This concept was introduced recently, and MAFLD's use in clinical practice needs further investigation (De et al. 2024).

The deposition of hepatocytes occurs when the breakdown of fat is surpassing. In the early stage of progression, it is connected with metabolic disorders like T2DM, obesity, dyslipidemia, and hypertension. The leading mechanism involves excessive fat accumulation in the liver, which is characterized by increased lipolysis in adipose tissues, activation of lipogenesis, and intake of a high‐fat calorie diet. This led to the progression of MAFLD (Ghazanfar et al. 2024). The beginning of MAFLD is distinguished by fat buildup in hepatocytes of 5% or more of liver weight. There is < 5% of fat deposition in healthy fat, mild fat accumulation is 5%–33%, moderate fat accumulation is 34%–66%, and severe fat deposition is more than 66% in the liver (Badmus et al. 2022). MAFLD is becoming a public health problem worldwide. Globally, the prevalence of MAFLD is increasing, and it is estimated that 32% of the adult population is affected by MAFLD. Additionally, the prevalence of obesity, diabetes, and aging is increasing rapidly, which contributes to an increase in MAFLD liver disease and mortality. There are two million deaths annually due to liver diseases, and it is accountable for 4% of all deaths. Around two‐thirds of all liver‐related problems happen in men (Peng et al. 2023). Increasing the metabolic risk factors such as obesity, hypertension, hyperglycemia, hypercholesterolemia, T2DM can contribute to high prevalence of MAFLD. Over the last two decades, these two metabolic disorders have contributed to the increasing highest prevalence of MAFLD in Asia. Overweight and insulin resistance are strongly linked with MAFLD (Ali et al. 2022). The prevalence of MAFLD in Pakistan is approximately 14%–30%, which differs by region. The main objective of this review is to assess the combined effects of flaxseed oil and olive oil on metabolic dysfunction‐associated fatty liver disease (MAFLD) and its associated complications. More specifically, the study aims to explore how the joint application of these oils can enhance lipid metabolism, decrease inflammation, and promote liver health, possibly offering an effective treatment strategy for managing MAFLD and reducing its negative health impacts.

## 2 Phytochemical Composition of Flaxseed Oil and Olive Oil

**FIGURE 1:**
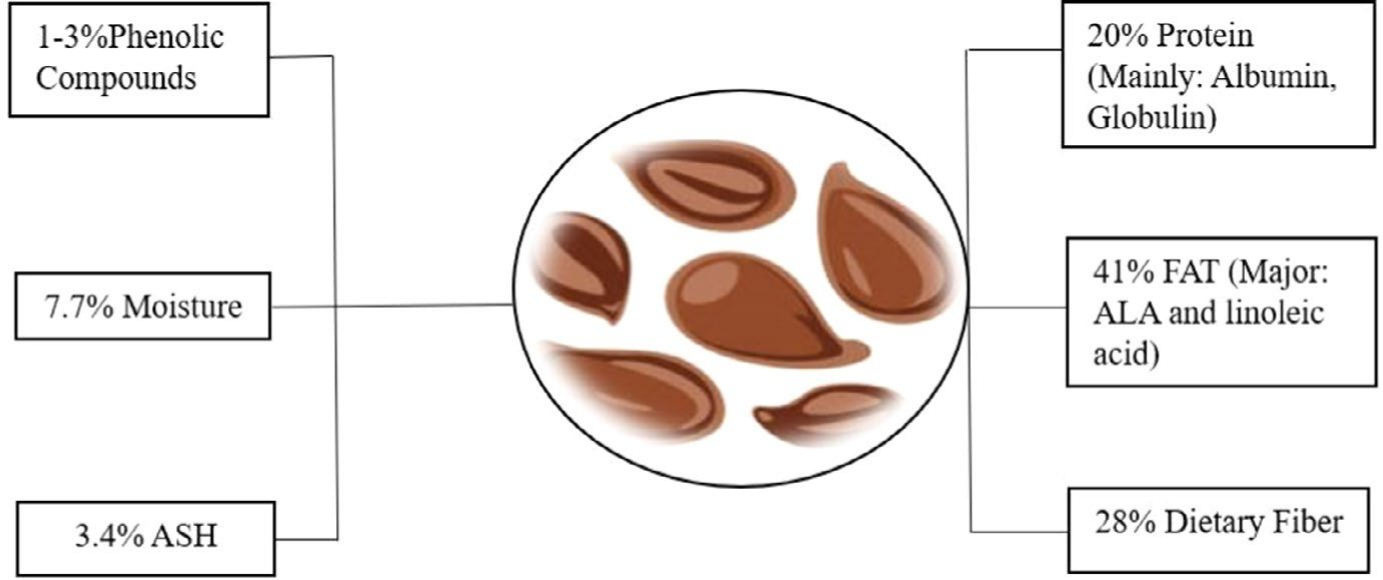
Phytochemical composition of flaxseed.

Olive oil was regarded as both a great diet and a medicinal agent by the Mediterranean people in antiquity. The health and nutritional benefits of olive oil have attracted much interest over the last 40 years (Aydin, Özcan, and Gümüş 2009). Some studies suggest a strong result of foods rich in monounsaturated fats, like extra virgin olive oil (EVOO), reducing liver fat and inflammation. Some reports present a decline in hepatic lipid accumulation using olive oil. Most studies focus on the consumption of olive oil, which is beneficial for the liver as it is a high source of monounsaturated fatty acid (Tedesco et al. 2023). Omega‐3 polyunsaturated fatty acids consist of many long‐chain fatty acids, such as αlinolenic acid (α‐ALA), eicosapentaenoic acid (EPA), docosapentaenoic acid (DPA), stearidonic acid (SDA), and docosahexaenoic acid (DHA) (Mantovani and Dalbeni 2021). Olive oil consists of Monounsaturated Fatty Acids (MUFA) specifically oleic acid which reduces fat accumulation in the liver and also contributes to insulin sensitivity regulation. EVOO contains some polyphenolic compounds (Table 1), such as oleuropein and hydroxytyrosol, which have anti‐inflammatory properties (Mitrovic et al. 2022). *Linum usitatissimum* mechanism of action and phytochemical composition provide overall benefits over other oils (Al‐Madhagy et al. 2023; Matthäus, Ozcan, and Al Juhaimi 2014). Flaxseed oil is rich in omega‐3 fatty acids, particularly α‐linolenic acid, which constitutes 40%–60% of its composition, following fish oil in abundance. Long‐chain n‐3 fatty acids provide health advantages similar to those of α‐linolenic acid in managing MAFLD. Compared to flaxseed, flaxseed oil is the most concentrated source of n‐3 fatty acids (Özcan et al. 2019; Mueed et al. 2022). Significant variations in the fatty acid makeup of the oils under study suggest that olive oil quality is influenced by varietal factors (Marongui et al. 2015; Ozcan et al. 2015). Flaxseed oil is enriched with omega‐3 fatty acids and is a good source of alpha‐linolenic acid (ALA), antioxidant lignans, and fiber. MAFLD has liver inflammation, oxidation, and fibrosis involved in its disease development, which are the exclusive characteristics of flaxseed that can overcome it. ALA has anti‐inflammatory properties, lignans are antioxidants, fiber lowers plasma lipid levels, and flaxseed can also reduce tissue fibrosis. This raises the possibility that flaxseed may benefit from an MAFLD model (Parikh et al. 2024). Flaxseed is abundant in various phytochemicals such as dietary fiber, phenolic compounds, protein, and many other components, as presented in Figure 1.

**TABLE 1:** Main phenols present in virgin olive oil.

Phenols	Range (mg/kg)
Hydroxytyrosol acetate	21.4–131.0
Hydroxytyrosol	0.0–25.4
Luteolin	0–10
Lignans	112–275
3,4‐DHPEA‐EA	25.6–310
Tyrosol derivative	0.0–113.4
4‐HPEA‐EDA	13.0–86.4
3,4‐DHPEA‐EDA	74.7–840
Ferulic acid	0.0–2.4
p‐Cumaric acid	0.04–0.6
Syringic acid	0.0–2.3
Caffeic acid	0.0–1.0
Vanillic acid	0.09–0.8
Tyrosol	0.10–123.1

## 3 Health Benefits of Flaxseed Oil and Olive Oil

Virgin olive oil is distinct from other vegetable oils due to its high concentration of certain phenolic compounds, which are responsible for its health advantages, along with its high unsaturated fatty acid content. The distinctive flavor and great durability of oxidation of virgin olive oil are also attributed to olive oil phenolics. Virgin olive oil's phenolic fraction is made up of a diverse range of substances, each with unique chemical characteristics and effects on the oil's quality (Dağdelen et al. 2013). Flaxseed oil and olive oil are both nutritionally rich oils that provide unique advantages for our health. Flaxseed oil is abundant in omega‐3 fatty acids, notably alpha‐linolenic acid (ALA), which is essential for diminishing inflammation, maintaining cardiovascular well‐being, and enhancing cognitive abilities (Table 2). Additionally, it promotes digestive well‐being, enhances skin moisturization, and maintains hormonal equilibrium. Conversely, olive oil is renowned for its abundant monounsaturated fats, including oleic acid, which promotes cardiovascular well‐being by reducing harmful cholesterol and elevating cholesterol levels. Olive oil has a high concentration of antioxidants, such as polyphenols, that safeguard cells from harm and possess potent anti‐inflammatory properties. In addition, olive oil promotes brain health, perhaps lowers the risk of cognitive decline and assists in regulating blood sugar levels. Some phenolics with significant antioxidant properties can be found in olive ( *Olea europaea* L.) fruits, oil, and derivatives (olive paste and table olive). Virgin olive oil (VOO) contains a lot of phenolics from several classes, including phenolic acids, phenolic alcohols, flavonoids, secoiridoids, lignans, and hydroxy‐isochrons (Uslu and Özcan 2022; Yorulmaz et al. 2012). Both oils enhance the health of the skin and hair, making them ideal supplements to a well‐rounded diet for general well‐being, as discussed in Table 3.

**TABLE 2:** Fatty acid profile of flaxseed oil.

Composition	Amount (%)	Description	Benefits	Reference
Alpha‐Linolenic Acid (ALA, Omega‐3)	50–60	A polyunsaturated essential fatty acid	Anti‐inflammatory characteristics; improves cardiovascular health and cognitive function while lowering the risk of chronic diseases	Singh et al. (2024)
Linoleic Acid (LA, Omega‐6)	12–18	A polyunsaturated essential fatty acid	Promotes skin health, regulates metabolism, and aids with immunological function	Kartikasari et al. (2024)
Oleic Acid (Omega‐9)	10–22	A monounsaturated fatty acid	Helps to maintain healthy cholesterol levels and promotes heart health	El‐feky et al. (2024)
Palmitic Acid	5–7	A saturated fatty acid	Provides energy, although excessive consumption may raise cholesterol levels	Beckett et al. (2024)
Stearic Acid	2–4	A saturated fatty acid	Considered neutral regarding cholesterol; supplies energy	Tan et al. (2024)

**TABLE 3:** Health advantages of flaxseed oil and olive oil.

Experiment	Results	References
20 g/day of flaxseed worked among patients with MAFLD for 12 weeks	After 12‐week intervention of flaxseed of total 235 patients, fatty liver grade and its biomarkers decrease with significant *p*‐value *p* < 0.001	Rezaei et al. (2020)
30 g/day of flaxseed worked among patients with MAFLD for 12 weeks	After 12‐week dietary intervention of flaxseed significant decrease in hepatic steatosis were observed *p* < 0.001 also change in metabolic profile and anthropometric measurements were shown	Yari et al. (2021)
32 g/day of olive oil worked among patients with MAFLD for 60 days	After 2‐month intervention of olive, there was significant reduction in fatty liver index with *p*‐value 0.004 and pro‐inflammatory cytokines also decreased	Patti et al. (2020)
> 37 g/day of olive oil worked among patients with MAFLD	The study included 2436 participants allocated into three groups. A group with MAFLD who received higher consumption of olive oil as compared to other groups, showed more significant results in reduction of hepatic biomarkers, lipid profile and diabetes with significant *p*‐value *p* < 0.001	Tedesco et al. (2023)
30 mL/day of olive oil worked among patients with cardiovascular disease for 12 weeks	A randomized controlled trial with 12‐week intervention of total 20 individuals who were divided in three different groups but the group with healthy diet plus 30 mL/day of olive oil showed better the diet quality and triglyceride‐glucose index with significant value *p* < 0.001	Dos Santos et al. (2022)
1000 mg/day of flaxseed oil worked among patients with coronary heart disease for 10 weeks	After follow‐up of 10‐weeks of total 120 patients who allocated into two groups but a group who received 1000 mg flaxseed oil showed better and significant reduction in serum insulin level, triglycerides. LDL cholesterol level and high‐sensitivity C‐reactive protein level	Jiang et al. (2022)
52 mL/day of olive oil worked among patients with overweight for 12 weeks	A total of 149 individuals were randomized into the three intervention groups, 50 participants in the EVOO group received 52 mL/day showed significant reduction inflammatory biomarkers and obesity	Longhi et al. (2021)
30 g/day of flaxseed worked among overweight patients for 12 weeks	A randomized controlled trial of total 60 participants enrolled and allocated into two groups. After 12 weeks of intervention significantly higher reduction in waist circumference and waist‐to‐hip ratio (both *p* < 0.05) in the flaxseed‐consuming group as compared with the control group	Ahmadniay motlagh et al. (2021)
25–50 mL/day of olive oil worked among patients with T2DM for 2 months	A total of 633 hypertensive patients were enrolled for 2 months of trial with (100 mg/day of oleuropein and 20 mg/day of hydroxytyrosol). Significant reductions (*p* < 0.0001) in triglycerides, fasting glucose and waist circumference	Hermans et al. (2020)
25 mL/day of olive oil worked among patients with dyslipidemic disease for 6 months	A clinical study of total 43 participants including 14 healthy, 12 dyslipidemic patients with hypercholesterolemia recently diagnosed, and 17 post‐infarct patients. All participants received (25 mL/day) for 6 months and showed significantly decrease in inflammatory markers	Zimmer et al. (2023)
30 g/day of flaxseed worked among patients with metabolic syndrome of 12 weeks intervention	A randomized controlled trial was conducted of total 70 patients. After 12‐weeks intervention, participants showed significant reduction in the serum concentration of insulin (*p* < 0.001), triglyceride (*p* = 0.001), total cholesterol (*p* < 0.001)	Morshedzadeh et al. (2021)
25 mL/day of flaxseed oil worked among patients with metabolic syndrome patients for 7 weeks	A total of 60 participants were allocated into two groups who received 25 mL/day flaxseed oil and showed serum IL‐6 levels significantly decreased with *p*‐value < 0.001 in the flaxseed oil group as compared to another group	Akrami et al. (2020)

## 4 Hepatoprotective Activity of Flaxseed and Olive Oil

**FIGURE 2:**
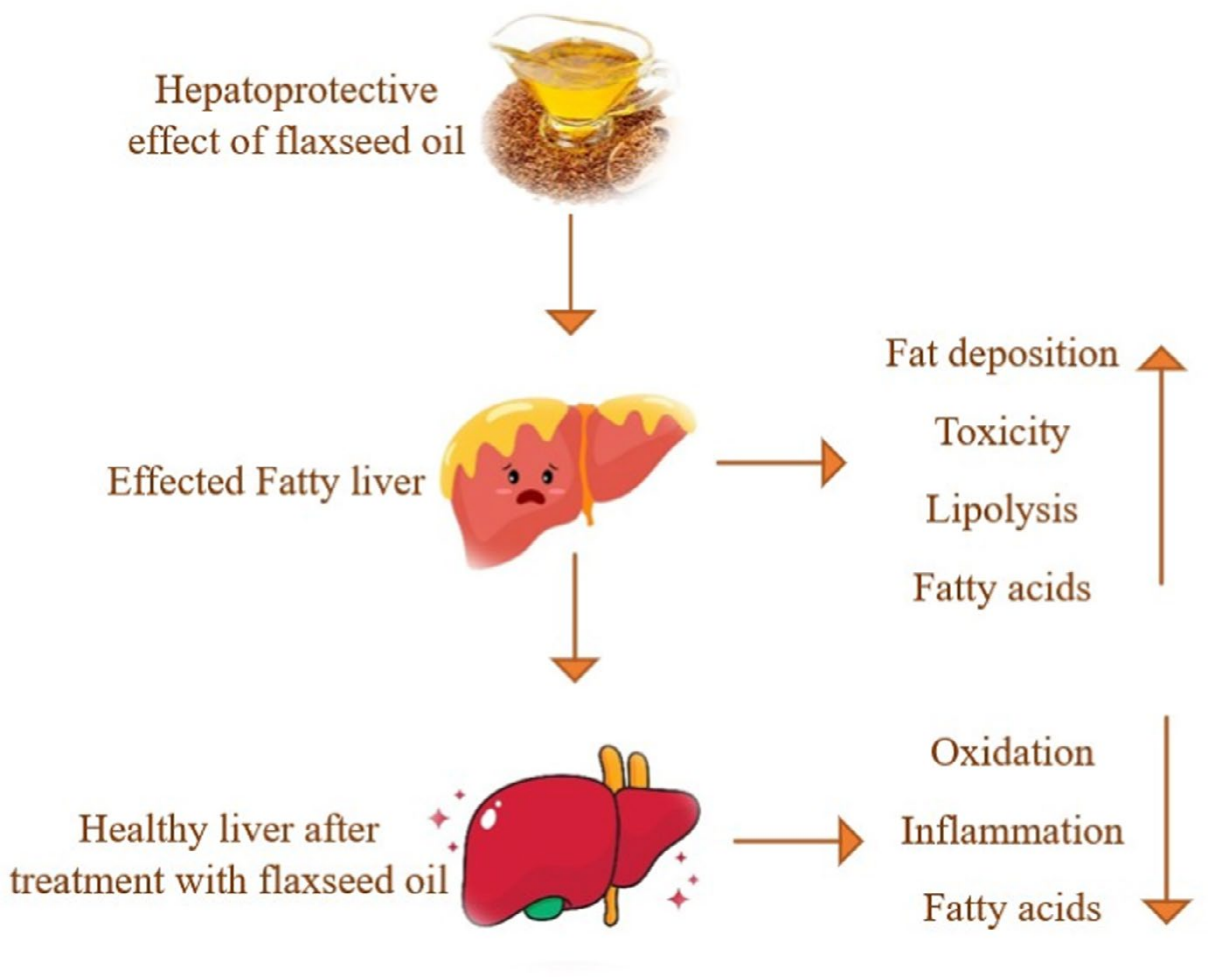
Hepatoprotective effect of flaxseed oil on inflammation, oxidation.

A randomized, double‐blind controlled trial examined the fact that omega‐3 fatty acids in flaxseed oil help maintain lipid metabolism and decrease fat deposition in the liver. Participants received flaxseed oil for 12 weeks and showed significant results in patients with MAFLD. The effect of α‐linolenic acid, lignans, and dietary fiber in flaxseed oil significantly decreases hepatic fat accumulation in patients. Altogether, these significant results prove that flaxseed oil has a strong beneficial ability to improve fatty liver in patients with MAFLD (Rezaei et al. 2020). These are the richest sources of niacin and vitamin E (tocopherol), which have strong antioxidant properties (Nowak and Jeziorek 2023). This comprehensive literature review mainly focused on flaxseed oil and the correlation between chemical composition and biological impact with other oil. In this review, the action mechanism of flaxseed and its phytochemical composition provide overall influences of flaxseed in contrast to other oils (Al‐Madhagy et al. 2023). The flax plants integrated the arrangement of biologically active compounds such as linosorbs (LO). LO's action mechanism is now illuminating the interaction of linosorbs with other active compounds present in flaxseed, which involve biological activity markers for various compounds (Shim et al. 2024). Due to flaxseed oil's anti‐inflammatory, antioxidant, anticancer, metabolic and cardio‐protective properties, it has a strong hepatoprotective effect compared to other PUFA‐rich oils, as shown in Figure 2.

This review assessed the effectiveness of olive oil on liver‐related indicators and showed highly significant results in reducing the risk of fatty liver diseases. Six randomized trials that used olive oil for cooking and eating explored potential effects on chronic liver disease (Ma et al. 2023). This study analyzed and evaluated the effect of consuming olive oil on diet and significantly reduced mortality of fatty liver diseases and metabolic syndromes (Bonfiglio et al. 2023). Some other important compounds such as cannabinoids, Aquamin, silymarin, and glutathione can lower the concentration of triglycerides. This study also modulates the activity of various components and many biochemical pathways involved in the improvement and management of MAFLD (Munteanu and Schwartz 2023). The diet's fat type significantly affects lipid peroxidation in patients with MAFLD. This study examined the effect of a hypocaloric diet including olive oil on hepatic steatosis and MAFLD. Olive oil contains monounsaturated fatty acids and omega‐3 fatty acids, which have more oxidation ability than saturated fats used in the diet. A diet containing olive oil significantly decreases cholesterol and triglyceride levels (Keshk et al. 2022).

## 5 Anti‐Inflammatory Activity of Flaxseed and Olive Oil

In addition to the fruit development stage and place of origin, the olive cultivar is thought to have a significant impact on a number of compositional characteristics of olive oil, including fatty acids, phenolics, and other elements that are all highly nutritious. Varietal variation may be the most important and determining factor for the oil fatty acid composition of various Tunisian cultivars among these several factors, according to reports (Özcan et al. 2019; Aljuhaimi et al. 2017). Flaxseed oil enriches with omega‐3 fatty acids and is a good alpha‐linolenic acid (ALA) source. ALA and lignans have anti‐inflammatory action, which raises the possibility that flaxseed may benefit in an MAFLD model (Parikh et al. 2024). Alpha‐linolenic acid (ALA) is a specific kind of omega‐3 fatty acid with anti‐inflammatory properties that minimizes inflammation by inhibiting inflammatory molecules like eicosanoids and cytokines, by lowering the inflammation, thus reducing hepatic cell damage and protecting from the progression of MAFLD. Lignans are a type of polyphenol that potentially lower inflammation by reducing the production of pro‐inflammatory cytokines (Ebrahimi et al. 2021). Omega 3 fatty acids in flaxseed oil have been shown to maintain lipid metabolism and reduce fat accumulation in patients with MAFLD. The benefits of *α*‐Linolenic acid present in flaxseed oil, a precursor of omega‐3 fatty acids, have strong benefits against fatty liver (Rezaei et al. 2020). Olive oil is the richest source of monounsaturated fatty acids, omega‐3 polyunsaturated fatty acids that stop liver fat accumulation by reducing the activity of hepatic lipogenesis. Olive oil contains some polyphenolic compounds, such as oleuropein and hydroxytyrosol, which have anti‐inflammatory properties and protect the liver from severe progression of MAFLD (Mitrovic et al. 2022). Foods rich in monounsaturated fats, like olive oil, reduce liver fat and inflammation. Some reports present a decline in hepatic lipid accumulation through olive oil. Most studies focus on the consumption of olive oil, which is beneficial for the liver as it is a high source of monounsaturated fatty acid (Tedesco et al. 2023). Oleocanthal (OC), a phenolic compound in olive, has anti‐inflammatory properties. Several studies suggest that long‐term use of olive oil in the diet protects the Mediterranean populations (Patti et al. 2020).

## 6 Antioxidant Activity of Flaxseed and Olive Oil

**FIGURE 3:**
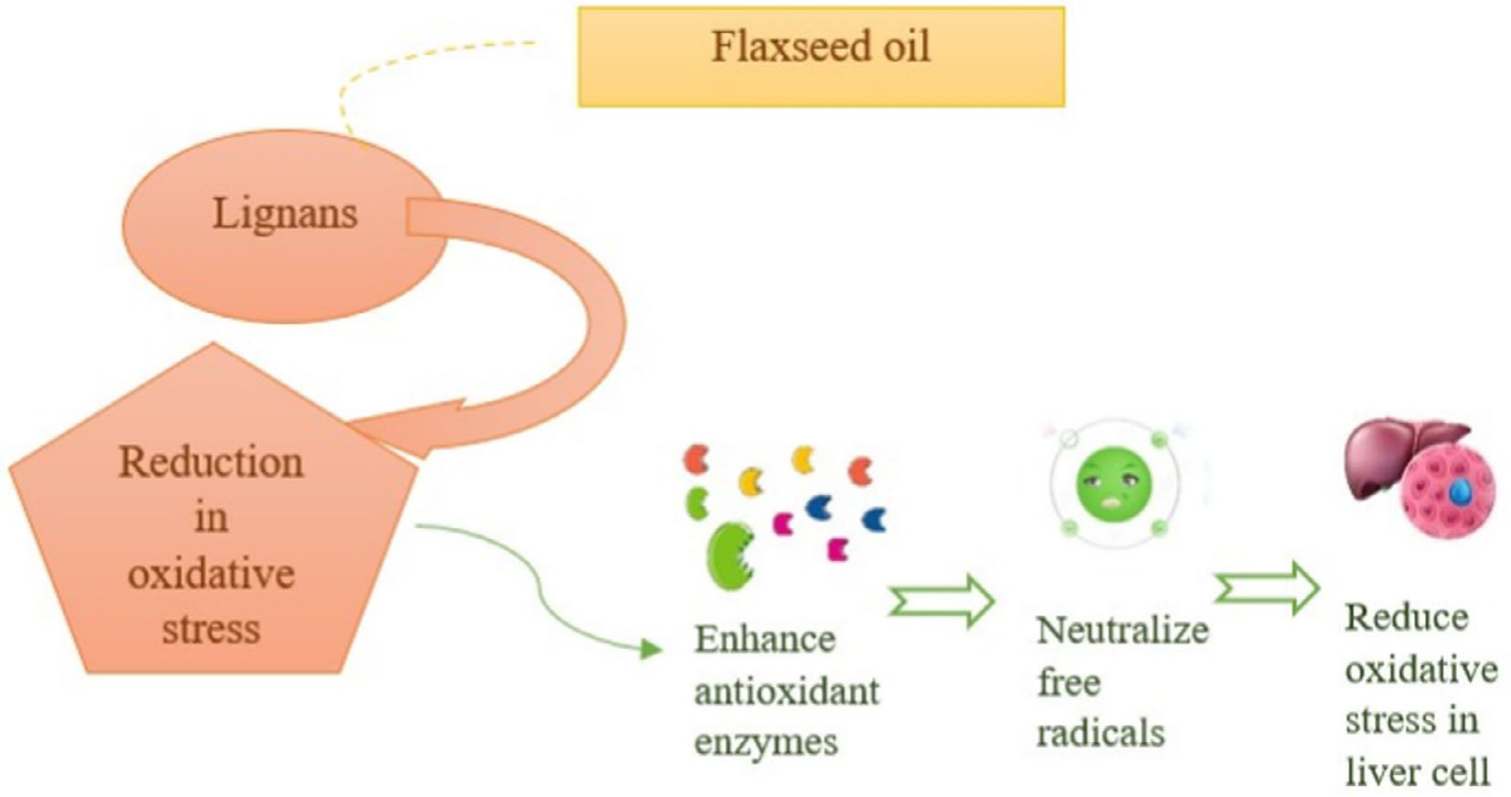
Mechanism action of flaxseed oil, lignans in antioxidation of liver cells.

Alpha‐linolenic acid (ALA) also has antioxidation properties that prevent liver cells from oxidative damage. ALA has anti‐fibrotic properties, which help MAFLD patients to get more severe forms of liver disease. Lignans, specifically secoisolariciresinol diglucoside (SDG), a polyphenolic compound, reduce the liver's inflammation and neutralize the free radicals. It also possesses the ability to reduce oxidative stress leading to the progression of MAFLD (Ebrahimi et al. 2021). Flaxseed oil consists of tocopherols, specifically gamma‐tocopherols, which have strong antioxidant properties that prevent hepatic cell damage by neutralizing free radicals. Phytosterols in flaxseed oil also have antioxidant activity and also lower cholesterol. It stabilizes hepatic cells by preventing them from oxidative damage (Ahmed and Fateh 2024). Lignan is an important component in flaxseed oil that is involved in the action mechanism of hepatic cell antioxidants, as prescribed in Figure 3.

Olive oil has many pro‐inflammatory and polyphenolic compounds with antioxidants that protect the hepatic cells from oxidative damage caused by the presence of free radicals (Mitrovic et al. 2022). Olive oil is highly enriched with bioactive compounds that can help manage MAFLD. Hydroxytyrosol, tyrosol, oleocanthal, oleuropein, and squalene are important compounds. Tyrosol works with hydroxytyrosol to prevent hepatic cell damage from oxidative stress and is involved in the antioxidant activity of olive oil. Squalene also has an antioxidant property that prevents lipid peroxidation. Olecanthal has antioxidant activity and oleuropein has anti‐fibrotic activity, preventing the liver from oxidative stress and extreme progression of MAFLD (Finicelli et al. 2021).

## 7 Cardio‐Protective Activity of Flaxseed and Olive Oil

**FIGURE 4:**
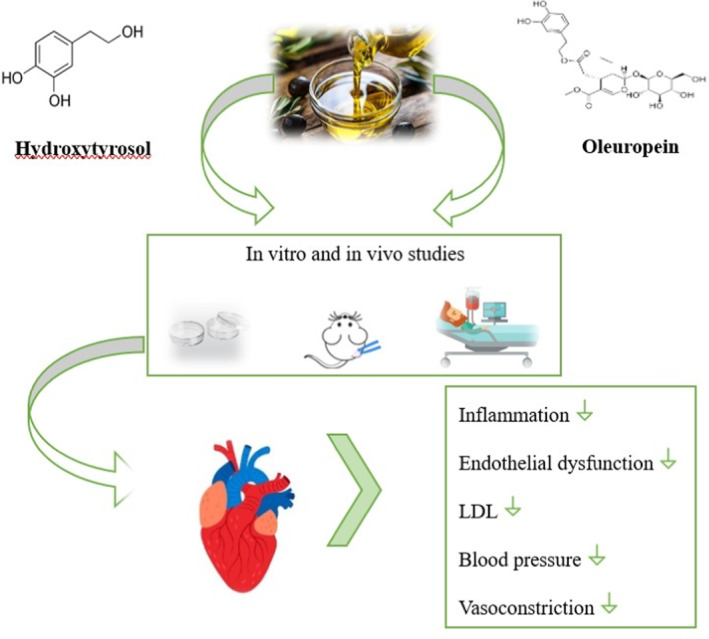
Effect of hydroxytyrosol and oleuropein on cardiovascular diseases, inflammation, low density lipoprotein.

Patients with disease MAFLD have more chances to develop cardiovascular diseases which increases the disease burden. Olive oil is enriched with components that have strong cardio‐protective functional activities, such as monounsaturated fatty acids particularly oleic acid exerting the properties of cardio‐protection (González‐Rámila et al. 2023). Olive oil has various polyphenolic compounds that contribute to cardio‐protective properties. For example, oleocanthal has anti‐inflammatory properties that help minimize inflammation and protect the heart from atherosclerosis. Another compound, tyrosol is involved in endothelial function and promotes healthy blood flow. It also prevents heart vessels from oxidative damage and reduces cholesterol oxidation. Thus, prevents plaque formation and supports a healthy cardiovascular system (Mehmood et al. 2020). Adding olive oil to the diet prevents hypertension at an early age. In MAFLD, there is a higher chance of developing heart disease as olive oil prevents cholesterol from oxidation. It has various components which significantly decrease the incidences of developing heart and chronic related diseases. In many studies, it has been shown that olive showed anti‐hypertensive and cardio‐protective effects. Phenolic compounds in olive oil are associated with positive effects on heart‐related disease biomarkers. Experimental studies demonstrated that the therapeutic influence of olive oil has exceptional benefits not only for cardiac health but also for other metabolic related diseases (AlMalki and Shahid 2020). A combined effect of hydroxytyrosol and oleuropein has a major impact on cardiac health, as shown in Figure 4.

**FIGURE 5:**
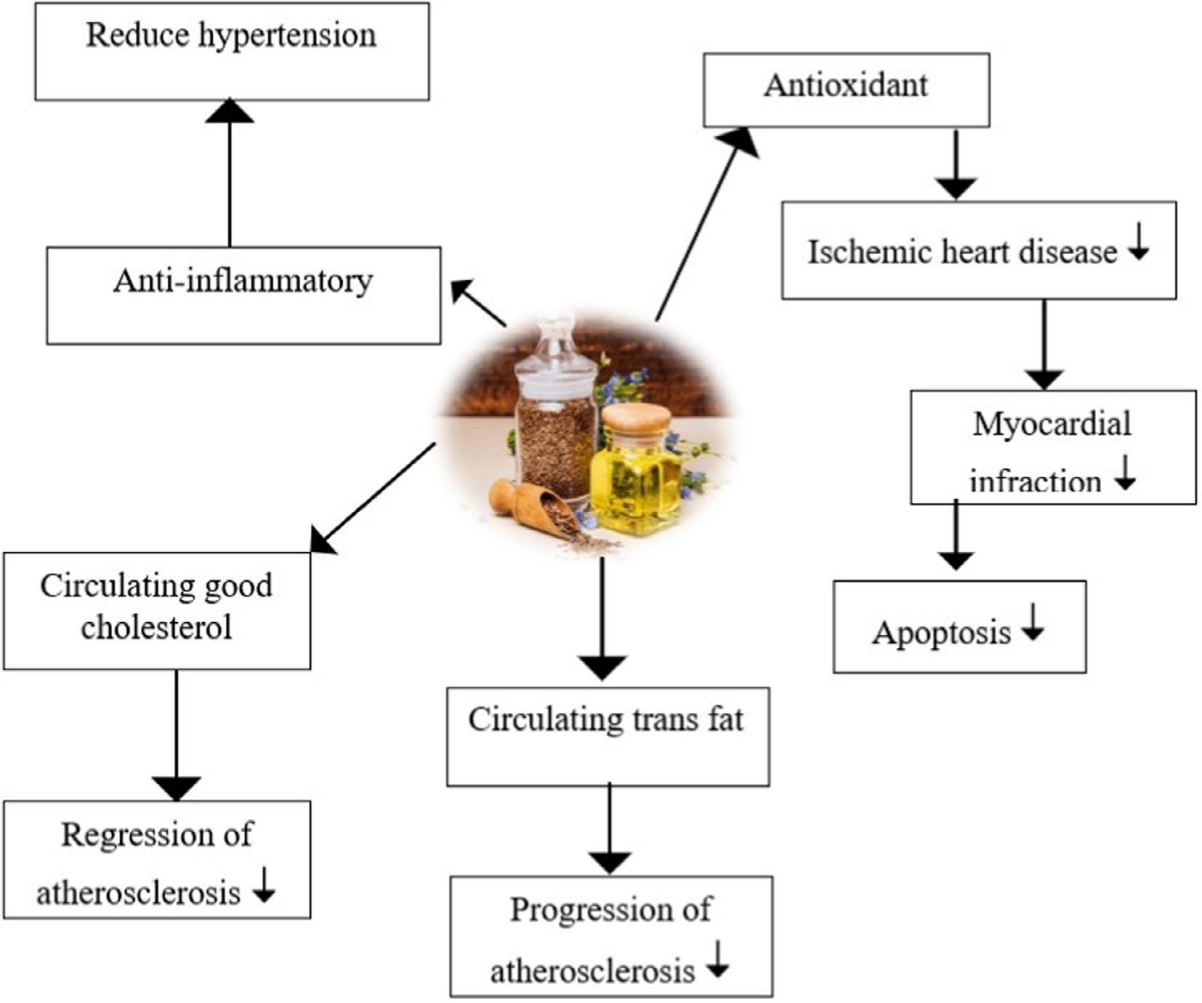
Cardio‐protective properties of flaxseed oil.

Inflammation is essential in cardiovascular diseases, and flaxseed oil has anti‐inflammatory compounds such as ALA, which help reduce inflammation. Lignans are involved in lowering LDL (bad cholesterol) and increasing HDL (good cholesterol) and contribute to reducing inflammation and oxidative stress. Phenolic compounds such as ferulic acid and p‐coumaric acid protect cardiovascular disease from oxidative stress (Prasad, Khan, and Shoker 2020). Among the components present in flaxseed oil is secoisolariciresinol diglucoside (SDG), which positively benefits heart health. It involves anti‐inflammatory and antioxidant effects, reducing total serum cholesterol and other atherogenic indices (Abdelwahab et al. 2023). Omega 3 and omega 6 fatty acids in flaxseed can benefit heart and lipids by improving lipid profile. They also have vasodilatory effects in suppressing inflammation, atherosclerosis, and other cardio complications. Fiber in flaxseed oil helps lower LDL cholesterol levels, improves blood lipid profile, and reduces the risk of cardiovascular disease. Flaxseed oil positively affects myocardial infarction by maintaining the gene expression that produces the cardio‐protective effect. Flaxseed oil is a rich source of plant‐based omega‐3 fatty acids, decreasing the chances of developing ventricular arrhythmias (Parikh et al. 2024). The action mechanism of major components present in flaxseed oil can potentially restore cardiac remodeling in heart toxicity. The study's findings demonstrated that using flaxseed oil improves cardiac functions, decreases apoptosis, lowers cholesterol levels, and decreases atherosclerosis progression (Boshra, Nazeam, and Esmat 2024). Flaxseed oil comprises many components that help in protection from innumerable cardiac health problems, as mentioned in Figure 5.

## 8 Diabetic Protective Activity of Flaxseed and Olive Oil

Diabetes contributes to the development of fatty liver diseases and its related complications. Adherence to olive oil is associated with lowering insulin sensitivity and regulating the glucose level. Olive oil contains several bioactive and polyphenolic compounds such as oleic acid, tocopherols, phytosterols, omega 3 fatty acids, tyrosol, hydroxytyrosol, and oleocanthal which have benefits to treat diabetes (Álvarez‐Amor et al. 2021). Olive oil consists of monounsaturated fatty acids (MUFA), specifically oleic acid, which regulates insulin sensitivity by enhancing glucose uptake by the cell. Hydroxytyrosol promotes proper pancreatic beta cell functioning and is involved in insulin sensitivity. Squalene and tocopherols, specifically alpha‐tocopherols, have antioxidant properties that prevent cell oxidation, which is commonly connected with diabetes (Dehghani et al. 2021). The therapeutic strategy of olive oil is linked with protection from the onset of T2DM and is capable of the restoration of proper functioning of beta cells. Beta cell dysfunction is the leading cause of diabetes, and monounsaturated fatty acid in olive oil is involved in metabolic control. Minor components in olive oil are statistically significant in lowering the risk of T2DM and its progressive complications (Marrano et al. 2021).

**FIGURE 6:**
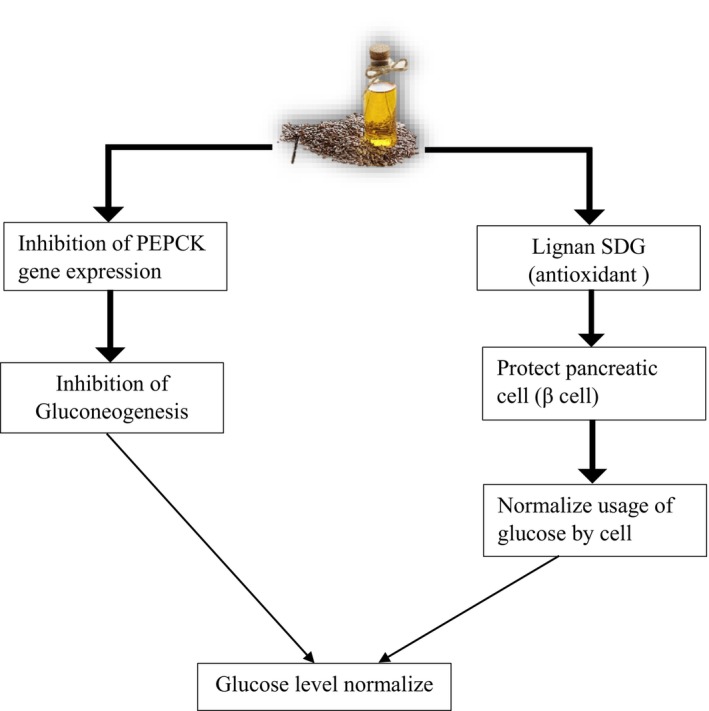
Effect of flaxseed oil on diabetes, inflammation, and serum glucose level.

Insulin resistance is the major cause of fatty liver; thus, both compounds help to improve insulin resistance. By lowering the insulin resistance, it improves the overall health. Flaxseed oil contains compounds that help improve insulin resistance, such as lignans, dietary fiber, ALA, magnesium, and phenolic acid (Hajiahmadi et al. 2020). The most specific lignans are secoisolariciresinol diglucoside (SDG), which is involved in improving blood glucose and insulin sensitivity. Flaxseeds contain both insoluble and soluble fiber, which regulate sugar levels and prevent many metabolic disorders. Lowering the insulin resistance it improves the overall health. Regular and proper intake of flaxseed oil in the diet can contribute to managing MAFLD (Ebrahimi et al. 2021). Phenolic and bioactive compounds present in flaxseed oil significantly regulate glucose and insulin metabolism. In flaxseed oil, polyunsaturated fatty acids (PUFA) are important in maintaining blood glucose levels. Oxidative stress is also involved in the progression of diabetes. SDG in flaxseed oil protects from oxidative damage in diabetes, ultimately preventing the liver from oxidative damage and worsening diabetes (Rehman et al. 2021). Flaxseed oil helps maintain serum glucose levels by acting on the antioxidant component lignan, specifically SDG and inhibits the gluconeogenesis pathway as prescribed in Figure 6.

## 9 Gastro‐Protective Activity of Flaxseed and Olive Oil

Various studies show that gut microbiota changes can affect NAFLD's incidence and expansion (Rong et al. 2023). Flaxseed oil enriches with omega‐3 fatty acids, promoting prostaglandin production, specifically prostaglandin E2 (PGE2). PGE2 helps prevent cells in the stomach lining as it has the cytoprotective ability; by enhancing the secretion of bicarbonate and mucus, it neutralizes stomach acid and prevents ulcers (Kręgielczak et al. 2023). Mucilage is a gel‐like fiber present in flaxseed oil. It forms a layer on the mucous membrane of the GI tract. This barrier prevents irritants such as pepsin, which can lead to ulcers. It also possesses a soothing ability that enhances GI tract healing (Manimurugan et al. 2023).

**FIGURE 7:**
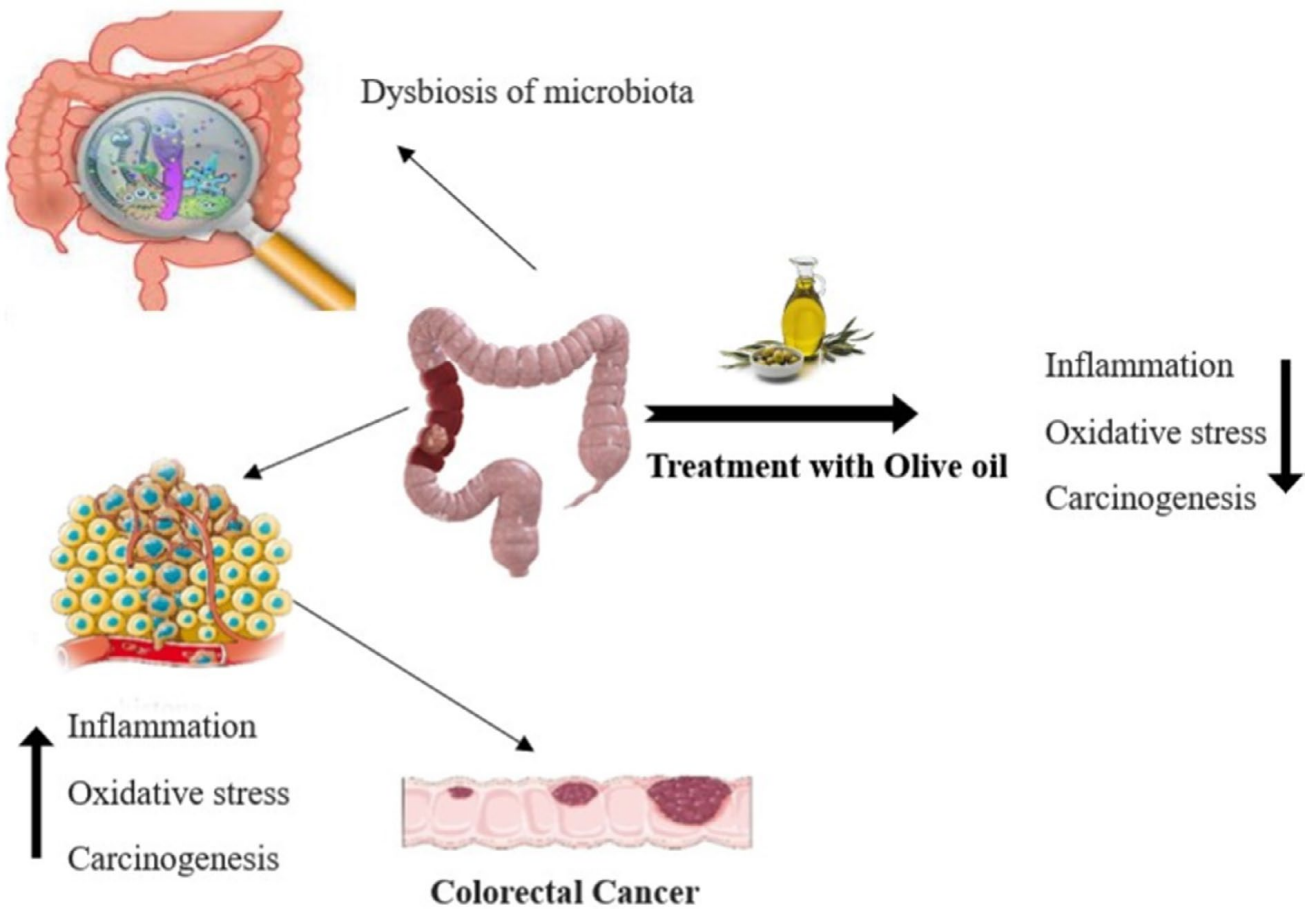
Implication of olive oil intake and inhibition of colorectal cancer, inflammation, oxidative stress.

Recent studies have discussed that flaxseed oil provides a positive impact and a favorable environment for good gut health bacteria. A healthy microbiome promotes gut health by balancing inflammatory response and digestive functions. It also reduces the risk of developing ulcers by regulating gastric acid secretions. Flaxseed oil's anti‐inflammatory and antioxidant properties help prevent and manage gut health‐related diseases and complications by modulating the production of inflammatory mediators and thus lowering inflammation (Mohamed and Farid 2024). Many studies show the importance of olive oil and the action of fatty acids to prevent gut health damage. Oxidative stress is the most common factor in gastric mucosal damage and ulcer progression. Antioxidants in olive oil, like oleuropein hydroxytyrosol, neutralize free radicals and play an important role in protecting against oxidative damage. Anti‐inflammatory compounds such as oleic acid inhibit pro‐inflammatory mediators like cytokines and thus prevent chronic inflammation. Olive oil is enriched with various monounsaturated fatty acids with anti‐inflammatory effects (Koc et al. 2020). Oleic acid can reduce pro‐inflammatory markers such as eicosanoids and cytokines and helps protect the GI from damage such as gastritis and ulcers. Olive oil encourages gastric mucus production, which protects the stomach lining from erosion and thus decreases the risk of ulcers. Also, antioxidants protect the stomach lining from cellular damage (Arismendi Sosa et al. 2022). Olive oil prevents the development of colorectal cancer (CRC) because (hydroxytyrosol and oleuropein) play an important role in reducing CRC's carcinogenesis, as shown in Figure 7.

## 10 Anti‐Obesity Activity of Flaxseed and Olive Oil

**FIGURE 8:**
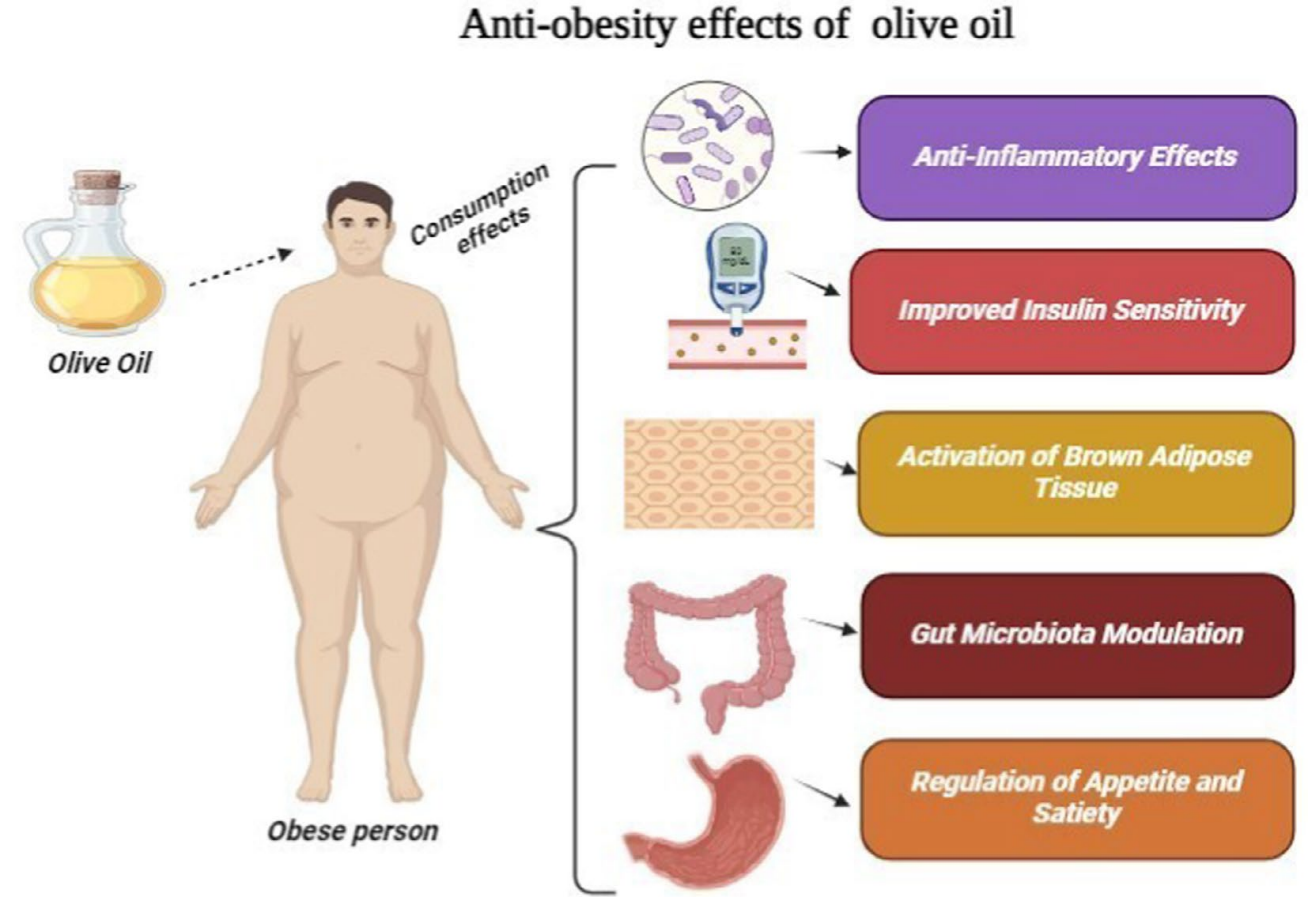
Anti‐obesity effects of olive oil.

The major cause of leading MAFLD is obesity, which contributes to its development and progression. Olive oil is highly enriched with bioactive compounds, providing exceptional benefits against obesity. Among these compounds, the pent acyclic triterpenic acids (oleanolic acid) have clinical relevance because of their major role of action mechanism in obesity prevention, vascular functions, insulin resistance, and associated complications (Claro‐Cala et al. 2022). Various polyphenols in olive oil contribute to weight management, mainly oleuropein and hydroxytyrosol, which have metabolic benefits and anti‐inflammatory properties as inflammation is associated with obesity. By lowering inflammation in the body can contribute to weight management. Oleic acid promotes glucose metabolism and helps maintain appetite. Olive oil also gives a feeling of fullness as it is a rich source of omega‐3 fatty acids (Hobani et al. 2022). Olive oil comprises such compounds that play a crucial role in activating Peroxisome Proliferator‐Activated Receptors Alpha (PPAR‐α). This receptor contributes to various bodily functions (Tahri‐Joutey et al. 2021). The components of olive oil, such as oleuropein and other polyphenols, involve activation of (PPAR‐ α), which causes fat metabolism and promotes lipolysis. It also involves burning calories by enhancing the activation of adipose tissues, which helps in weight loss, as mentioned in Figure 8.

Flaxseed oil contains both insoluble and soluble fiber, which contribute to decreasing cholesterol and triglyceride levels. This fiber also regulates sugar levels and prevents many metabolic disorders. Fiber also encourages the feeling of satiety and contributes to minimizing calorie intake. Altogether, these compounds help in weight management. Omega‐3 fatty acids in flaxseed oil help to reduce fat storage in the body. ALA influences fat metabolism by encouraging lipolysis and thus decreases the deposition of fat in the liver (Raole and Raole 2022). Adiponectin is an adipokine released by adipose tissue regulated by omega‐3 fatty acids. It plays an important role in the metabolism of adipose tissues by decreasing fatty acid synthesis. Intake of flaxseed oil in the diet increases adiponectin expression in visceral fat and helps control obesity and thermogenesis. Omega‐3 fatty acids help suppress obesity and prevent various metabolic‐associated diseases and complications (Seike, Ashida, and Yamashita 2024).

## 11 Anti‐Microbial Activity of Flaxseed Oil and Olive Oil

Any microbial agent present in gut microbiota causes disturbance in gut health. As gut health is linked with the liver through the portal vein, disturbance or gut complications can contribute to liver issues. Fatty acids in flaxseed oil, particularly ALA, can potentially have an anti‐microbial effect by disrupting microbial membranes. This led to the leakage of cellular material and thus became the cause of cell death. In this way, fatty acids prevent microbes and provide effective anti‐microbial properties. Lignans have both antioxidant and anti‐inflammatory properties and protect microbes by inhibiting the growth of many bacteria that interfere with cellular activities (Cacciatore et al. 2022). Phenolic compounds in flaxseed oil also process anti‐microbial activity by inactivating the enzymes and microbial cell membranes. They have effects against various pathogens that can cause diseases. Cyclic peptides also present in flaxseed oil, called cyclolinopeptides, have strong anti‐microbial activity due to their unique structure and ability to defend against microbes (Liu et al. 2022). Olive oil has many polyphenolic compounds and monounsaturated fatty acids, which exhibit strong anti‐microbial activity. Oleuropein can break the cell wall of the microbe and cell lysis. Other hydroxytyrosol and tyrosol compounds inhibit pathogens' growth and progression by damaging their cell membrane. Oleic acid also has the potential to disturb microbial cells and inhibit their formation (Bensehaila et al. 2022).

## 12 Conclusion

The current study concluded that the incorporation of flaxseed oil and olive oil into the diet has various positive and beneficial impacts on MAFLD and many other metabolic complications. Many pharmacological effects, including anti‐inflammatory, antioxidant, antibacterial, anti‐obesity and antidiabetic effects of flaxseed oil and olive oil, have been demonstrated in this study. Multiple bioactive and phenolic compounds make them more potent to protect against various diseases. They also contribute to several nutritional advantages. This current research may help understand the therapeutic and effective mechanism of components present in flaxseed and olive oil, which are beneficial in treating various health issues. Combining these two oils in a balanced diet can be an effective strategy against managing MAFLD alongside other health benefits.

## Author Contributions

**Sana Noreen:** project administration (equal), writing – original draft (equal), writing – review and editing (equal). **Bushra Hashmi:** investigation (equal), resources (equal), visualization (equal), writing – review and editing (equal). **Tabussam Tufail:** conceptualization (equal), investigation (equal), methodology (equal). **Ali Ikram:** data curation (equal), formal analysis (equal), supervision (equal). **Muhammad Tayyab Arshad:** validation (equal), visualization (equal), writing – review and editing (equal). **Kodjo Théodore Gnedeka:** conceptualization (equal), investigation (equal), writing – original draft (equal).

## Consent

The authors have nothing to report.

## Conflicts of Interest

The authors declare no conflicts of interest.

## Institutional Review Board Statement

This study did not involve humans or animals.

## Data Availability

The data supporting this study's findings are available from the corresponding author upon reasonable request.
